# Ligamentoplastie du ligament croisé antérieur du genou chez le sportif: étude rétrospective à propos de 80 cas dans le Département d’Orthopédie de Tataouine, Tunisie

**DOI:** 10.11604/pamj.2020.36.2.22880

**Published:** 2020-05-04

**Authors:** Mourad Hammami, Nizar Sahnoun

**Affiliations:** 1 Service de Chirurgie Orthopédique et Traumatologie, Hôpital Tataouine, Tataouine, Tunisie; 2 Service de Chirurgie Orthopédique et Traumatologie, Centre Hospitalier Universitaire Habib Bourguiba, Sfax, Tunisie

**Keywords:** Ligamentoplastie, arthroscopie, sportif, Ligamentoplasty, arthroscopy, athlete

## Abstract

La ligamentoplastie du ligament croisé antérieur a pour but de corriger la laxité antérieure du genou afin de retrouver une stabilité et une indolence. Le but de notre travail est d'évaluer les résultats fonctionnels à court et à moyen terme de notre série. C'est une étude rétrospective réalisée au service de Traumato-Orthopédie de l'hôpital de Tataouine au Sud tunisien, étalée sur une période de 5 ans, allant du mois de janvier 2013 au mois d'avril 2018, concernant 80 sportifs tous présentant une laxité chronique du genou secondaire à une rupture du LCA suite à un accident sportif. Tous les patients ont été opérés par le même chirurgien sous arthroscopie, le transplant a été prélevé soit du tendon rotulien soit des tendons de la patte d'oie. L'évaluation fonctionnelle a été faite selon le score fonctionnel de Lyshlom-Tegner. Dans notre étude, la reconstruction du LCA par le tendon rotulien a été pratiquée chez 20 patients, et par les tendons de la patte d'oie pour 60 patients. Le délai moyen de la reprise du sport dans notre série était de 9 mois. L'échelle de l'activité de Tegner a montré que 65 patients ont repris le sport au même niveau, avec un délai moyen de 9 mois, avec une reprise du sport à un niveau inférieur pour le reste. La ligamentoplastie du ligament croisé antérieur sous arthroscopie avec une maîtrise de la technique et une rééducation post opératoire donnent des résultats fonctionnels satisfaisants avec reprise de l'activité sportive.

## Abstract (en)

Ligamentoplasty of the anterior cruciate ligament is performed to correct the anterior laxity of the knee and to regain stability and indolence. The purpose of our study was to assess short-term and medium-term functional outcomes in our case series. We conducted a retrospective study in the Department of Orthopedics and Traumatology at the Hospital of Tataouine in the South of Tunisia over a period of 5 years, from January 2013 to April 2018. The study involved 80 athletes with chronic laxity of the knee, secondary to a fracture of the anterior cruciate ligament caused by sport accident. All patients were operated by the same surgeon. Treatment was based on arthroscopic reconstruction using patellar tendon or goose foot. Functional assessment was based on Lyshlom-Tegner functional score. In our study, reconstruction of the anterior cruciate ligament using patellar tendon was performed in 20 patients, while in 60 patients using goose foot. The average time of resumption of sport activity in our series was 9 months. Tegner Activity Scale showed that 65 patients resumed the same level of sport, with an average time of restoration of 9 months. The other patients resumed a lower level of sport. Ligamentoplasty of the anterior cruciate ligament performed by an arthroscopic surgery specialist and post-operative rehabilitation provide satisfactory functional results, with resumption of sport activity.

## Introduction

La rupture du ligament croisé antérieur (LCA) du genou est l'une des lésions ligamentaires du genou les plus fréquemment rencontrées, touche essentiellement la population jeune pratiquant des sports de pivot et de contact [1]. La ligamentoplastie du LCA a pour but de corriger la laxité antérieure afin de retrouver un genou stable, indolore et fonctionnel, permettant la reprise de l'activité antérieure, de façon durable, tout en limitant les lésions dégénératives [2]. Le but de notre travail est d'évaluer les résultats fonctionnels à court et à moyen terme de notre série.

## Méthodes

**Figure 1:**
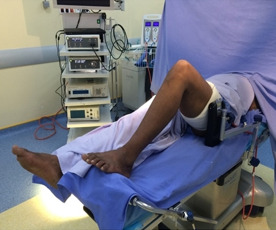
Installation sur table: genou fléchi à 90° avec un appui placé au bout de la table

**Figure 2:**
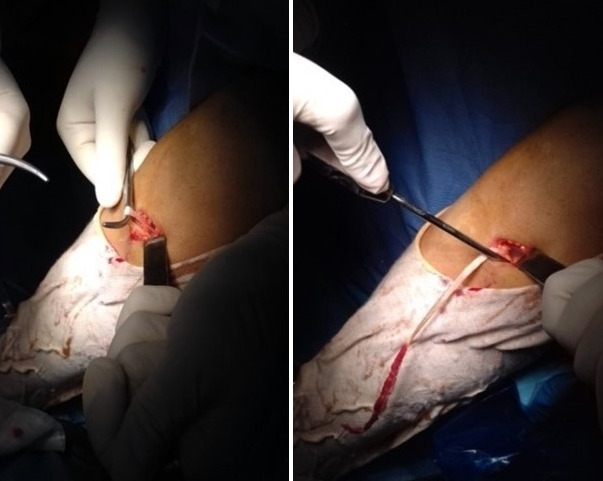
Prélèvement du tendon droit interne et du semi-tendineux

**Figure 3:**
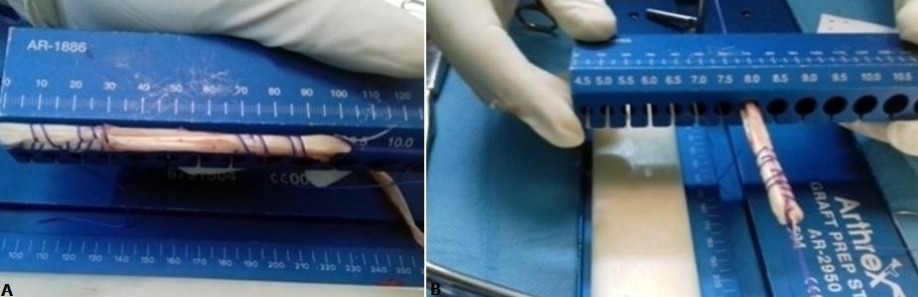
Mesure de la longueur du greffon (A) et calibrage du greffon type DIDT (B)

**Figure 4:**
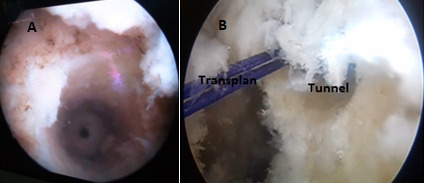
Aspect du tunnel fémoral sous arthroscopie après forage (A), passage du transplant dans le tunnel fémoral (B)

**Figure 5:**
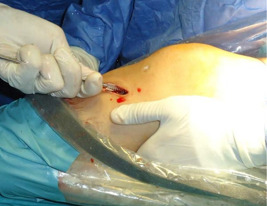
Fixation tibiale du transplant par des vis d’interférence résorbables

C'est une étude rétrospective réalisée au service de Traumato-Orthopédie de l'hôpital de Tataouine au Sud tunisien, étalée sur une période de 5 ans, allant du mois de janvier 2013 au mois d'avril 2018, et concernant 80 patients tous présentant une laxité chronique du genou secondaire à une rupture du LCA. Les critères d'inclusion sont les sujets sportifs présentant une rupture du LCA et confirmé par l'IRM du genou. Tous les patients ont été opérés par le même chirurgien. La totalité des patients ont été opérés sous arthroscopie en utilisant la technique de Kenneth Jones(KJ)ou la technique prélevant le greffon du droit interne et du demi tendineux (DIDT), l'installation utilisé est le décubitus dorsal genou fléchi à 90° avec un appui placé au bout de la table (Figure 1), on a procédé au prélèvement du greffon selon le site donneur (Figure 2) avec un temps de préparation et de calibrage (Figure 3) et après on a procédé au forage du tunnel fémoral borgne de dedans en dehors puis tibial de façon indépendante du tunnel fémoral (Figure 4) et tibial avec passage du transplant et sa fixation par des vis d'interférence (Figure 5). Toute lésion méniscale associée responsable de blocage du genou a été traitée par méniscectomie partielle. Les données des patients ont été recueillies sur une fiche de renseignement concernant l'âge le sexe les antécédents personnels et le type de sport pratiqué. L'évaluation fonctionnelle a été faite selon le score fonctionnel de Lyshlom-Tegner. L'ensemble des données ont été saisies et analysées au moyen de logiciel SPSS. Les variables qualitatives ont été décrites par les moyennes alors que les variables quantitatives ont été décrites par les effectifs et les pourcentages.

## Résultats

L'âge moyen de notre population est 27,3 ans, notre série comporte 76 hommes et 4 femmes. Vingt (20) patients étaient des sportifs compétitifs le reste sont des sportifs de loisir. La reconstruction du LCA a été réalisée par le tendon rotulien (KJ) chez 20 patients, et 60 patients étaient opérés selon la technique de reconstruction par DIDT. La durée moyenne d'hospitalisation était de trois jours. Le délai moyen de la reprise du sport dans notre série était de 9 mois. Nous avons noté deux cas d'hémarthrose (3% des patients), une hyposthésie de la jambe était notée chez cinq patients (6% des cas). Un ressaut rotatoire tous après DIDT chez six patients, un syndrome d'algodytrophie dans quatre cas, une rupture du greffon dans deux cas après DIDT un seul cas de raideur du genou d'après KJ et un seul cas du flessum du genou secondaire (syndrome de cyclope), et un seul cas de récidive de lésion méniscale. Le recul postopératoire dans notre série était en moyenne de 30 mois. Soixante patients soit 75% des cas étaient très satisfaits du résultat opératoire. Quant à l'échelle de l'activité de Tegner, 65 cas ont repris le sport au même niveau avec un délai moyen de 9 mois, avec une reprise du sport à un niveau inférieur pour le reste.

## Discussion

Nos résultats selon l'échelle de Lyshlom-Tegner, sont comparables avec certaines séries de la littérature [3-5] (Tableau 1). Concernant les particularités techniques: pour le choix du transplant, DIDT était le greffon de choix dans notre série, utilisé dans 75% alors le tendon rotulien a été réservé chez les sportifs compétitifs. Cooper *et al.* [6] les deux greffes se valent en ce qui concerne leurs excellentes propriétés biomécaniques, le test de lachman, la laxité, la mobilité, la fréquence de ré-ruptures, et le score fonctionnel. A noter qu'il y a une supériorité du tendon rotulien en ce qui concerne le ressaut et le retour au sport par rapport au DIDT, mais cette dernière donne moins de douleurs résiduelles [7-8]. Le forage du tunnel fémoral reste un point de discussion, dans notre série, nous avons fait un forage du tunnel fémoral borgne de dedans en dehors du fait du gain de durée opératoire et de la réalisation d'une seule incision. Cambât *et al*. [9] comme Panni *et al.* [10] défendent la technique de dehors en dedans, pour réaliser un tunnel fémoral tel que son bord antérieur se situe au niveau du point isométrique [9-10]. En effet le positionnement fémoral du transplant influence son isométrie [11]. Pour la fixation du transplant La solidité de la fixation dépend du transplant mais également du moyen de la fixation utilisé [12]. Les deux principaux modes de fixation sont les vis d'interférence et la fixation corticale par l'endobouton et apparentés [13]. Pour le tendon rotulien (KJ), la fixation par vis d'interférence en tibial et en fémoral reste l'indication de choix alors que pour le DIDT, les vis peuvent être utilisées et présentent moins de résistance et un risque de glissement plus important, notamment au versant tibial, en raison d'une densité osseuse moindre du tibia. Il est donc recommandé d'utiliser des vis de plus grand diamètre mais aussi plus longues pour le fémur [14]. Sur le versant fémoral de la greffe, la fixation par les endoboutons reste le meilleur choix mais peut exposer le transplant à l'élongation (*Bungee effect*) ou à l'élargissement du tunnel (effet essuie-glace) [15].

**Tableau 1:** Résultats fonctionnels dans la littérature

	Technique	Nombre de patients	Recul en Mois	Lysholm	Tegner
Potel JF *et al* SFA KJ 1999 [3]	KJ	655	18	-	-
Potel JF *et al* SFA DIDT 1999 [3]	DIDT	403	18	-	-
Jaeger *et al* 2002 [4]	Mac-Intosh	94	66	93,2	6,7
OKSMAN *et al* 2003 [5]	Fascia lata renforcé au gracile	60	30	96	7
Notre série 2018	KJ + DIDT	80	26	81	-

## Conclusion

La maitrise de la technique chirurgicale de ligamentoplastie et la rééducation post opératoire permettent d'avoir des résultats fonctionnels satisfaisants.

### Etat des connaissances actuelles sur le sujet

- Les ruptures du ligament croisé antérieur sont fréquentes chez le sportif;
- La ligamentoplastie du LCA est le traitement de choix des ruptures du LCA;
- Le résultat fonctionnel chez le sportif conditionne sa reprise de son activité sportive.

### Contribution de notre étude à la connaissance

- La maîtrise de la technique chirurgicale et l'un des facteurs qui permettent d'avoir de bons résultats fonctionnels;
- La rééducation post-opératoire immédiate nous a permis d'avoir des résultats fonctionnels satisfaisants;
- Le sportif peut garder un ressaut rotatoire surtout après une ligamentoplastie type DIDT.

## Conflits d’intérêts

Les auteurs ne déclarent aucun conflit d'intérêts.
